# Clinical evaluation of thumb base osteoarthritis: A scoping review

**DOI:** 10.1177/17589983211002560

**Published:** 2021-03-21

**Authors:** Mirka Normand, Tiffany S Tang, Jean-Michel Brismée, Stéphane Sobczak

**Affiliations:** 1 Département d'anatomie, Université du Québec à Trois-Rivières, Trois-Rivières, Canada; 2 Chaire de recherche en anatomie fonctionnelle, Université du Québec à Trois-Rivières, Trois-Rivières, Canada; 3 Rehabilitation Department, Pequot Health Center, Yale New Haven Health System, Groton, CT, USA; 4 Physical Medicine and Rehabilitation, California Pacific Medical Center, San Francisco, CA, USA; 5 Department of Rehabilitation Sciences and Center for Rehabilitation Research, School of Health Professions, Texas Tech University Health Sciences Center, Lubbock, TX, USA

**Keywords:** Clinical examination, functional assessment, osteoarthritis, thumb base, trapeziometacarpal joint

## Abstract

**Introduction:**

Thumb base osteoarthritis (OA) is a prevalent hand OA phenotype, associated with specific risk factors, treatment strategies, and requiring a distinct subset of evaluative approaches. This paper aimed at surveying our clinical evaluative methods and identifying gaps in our ability to capture the thumb’s unique attributes and how they could impact our treatment recommendations.

**Methods:**

A scoping review was conducted in accordance with the Joanna Briggs Institute methodology to gather relevant published and non-published articles regarding clinical tests currently available to assess the physical presentation of thumb base OA with special consideration of its specific multifactorial parameters namely architecture, ligaments, biomechanics, neuromuscular control, and proprioception. A full search strategy of MEDLINE, CINAHL, EMBASE, Scopus, Google Scholar, and Clinical Trials.gov from their inception through May 2020 was performed.

**Results:**

Of 1936 citation identified, 54 met the inclusion criteria. Fifty-two clinical physical tests for the evaluation of thumb base OA were extracted, most of which well suited to address research questions regarding efficacy of clinical intervention, however providing limited information regarding the underlying impairments of ligaments, biomechanics, neuromuscular or proprioceptive components.

**Conclusions:**

The tests and measures specific to the basal thumb OA phenotype, and capable of isolating its multifactorial contributors are scarce. Our limited physical assessment repertoire impedes our ability to describe and answer explicative research questions. Without these we cannot evaluate the effect of conservative management and provide specific treatment recommendations. Further research is needed to develop and validate distinct clinical tools for this debilitating pathology.

## Introduction

Thumb base osteoarthritis (OA) is a prevalent distinct hand OA subset that results in considerable clinical burden.^1^ Hand OA encompasses three different phenotypes (thumb base OA, nodal OA, erosive OA) each associated with different risk factors requiring different treatment strategies.^2^

The risk factors for thumb base OA include mechanical load, hypermobility, systemic factors such as obesity, hormones and other yet unclear potential metabolic contributors, as well as genetic influence.^1,3–5^ Although the clinical diagnosis of OA may be reached in the at-risk population using the classification criteria for OA of the hand developed by the American College of Rheumatology (ACR),^6,7^ the classification does not distinguish between OA in interphalangeal and thumb base joints.^1^

The thumb's broad range of performance from strong grip to very precise manipulations is a testimony to its complex nature. With its kinematic structure organized in a serial chain of five, invariant, nonorthogonal and nonintersecting rotational degrees of freedom (one at the interphalangeal joint, two at the metacarpophalangeal joint and two at the carpometacarpal joint), nine activating muscles (5 intrinsics, 4 extrinsics), and elaborate neuromuscular and proprioceptive network, it translates into a diverse crowd of kinematic model types affecting its functionality,^8,9^ which challenges our understanding of its pathomechanics.

Several recent systematic and scoping reviews have shown that the ability to effectively isolate and assess the efficacy of rehabilitative interventions for thumb base OA is limited.^10–14^ Despite confirmation of improvement in pain and function, their conclusions regarding the effect of non-surgical therapies for the management of OA of the hand and thumb remain unable to provide specific treatment guidance due to the heterogeneity of the study designs and outcome parameters.

Patient selection of many rehabilitation clinical trials is based on the radiographic disease state through different grading classifications of articular degeneration. A systematic review^15^ and recent primary research study^16^ on the OA classification's inter and intra-rater reliability found that the Eaton method had low to moderate reliability, suggesting that it should be used with caution when correlating with clinical findings and making treatment decisions. The authors propose that other qualitative factors, such as reported symptoms and physical examination are more important for choosing treatment modalities than Eaton radiographic classification. Although the Osteoarthritis Research Society International (OARSI) identified some core outcome measurement sets for studies in hand OA and a list of methods and instruments that should be used to measure symptoms or structures,^7^ to our knowledge no recommendations have yet been proposed in consideration of the recognized multifactorial nature of thumb stability and mobility. The existence of a broad variety of presentations of thumb kinematic models, resulting from its multifactorial nature,^8,9^ requires the establishment of an expended evaluative approach considering its unique characteristics; architectural, ligamentous, biomechanical, neuromuscular, and proprioceptive.^17^

Another explanation for the limited treatment guidance of conservative management of thumb base OA could reside in the selection of rehabilitation trial designs, perhaps leaning more toward the pragmatic versus explanatory side of the design continuum, where the primary outcome although meaningful, may not be the direct result of the intervention, as described in the OARSI clinical trials recommendations for rehabilitation intervention for OA.^18^ For example, the benefits of the use of a thumb orthosis may be measured through functional performance or with a patient-reported outcome measure, which makes it difficult to determine the ideal type of orthosis, as the primary outcome measure does not target the underlying aspects of the intervention such as the biomechanical rationale. To move the research efforts towards the explanatory end of the spectrum, we propose to refine the physical function and hand strength domains and associated outcome measures specific to the thumb base OA phenotype, looking at clarifying the presentation of the problematic thumb chain.

Current statistics on occurrence of thumb base OA in males and females in their fifth and sixth decade of life reaches 11% and 33%, respectively.^19^ As the American population above the age of 65 is projected to increase from 16% to 22% by 2050,^20^ the expected influx of incidences compels us to revise our approach to the assessment of thumb base OA, as a better definition of the problem could help lay the foundational blocks of preventative treatment.

A preliminary search of PROSPERO, MEDLINE, the Cochrane Database of Systematic Reviews, and the Joanna Briggs Institute (JBI) Database of Systematic Reviews and Implementation Reports was conducted and no current or underway systematic reviews on the topic were identified.

The objective of this scoping review was to map the current evidence on clinical physical tests to assess thumb base OA and provide an analysis of their coverage of the multifactorial components specific to the thumb. The review aims to address the following questions: i) what are the current clinical physical tests of thumb base OA; ii) which thumb dimensions are considered by current tests; and iii) what are the gaps in the approach to assessment of the multifactorial nature of thumb OA?

## Methods

This scoping review was conducted in accordance with the Joanna Briggs Institute methodology.^21,22^ The Institute and its international collaborating entities promote and support the synthesis, transfer and implementation of evidence through identifying feasible, appropriate, meaningful and effective healthcare practices to assist in the improvement of healthcare outcomes globally.^23^ Scoping reviews, like systematic reviews focus on rigor, reproducibility, and transparency. They draw on evidence from any research methodology and may also include evidence from non-research sources. In this manner, scoping reviews provide a comprehensive overview to address broader review questions than traditionally more specific systematic reviews of effectiveness or qualitative evidence. Scoping reviews are particularly helpful when the literature is complex and heterogeneous,^21^ as is the case regarding clinical tests for thumb base OA.

### Inclusion criteria

#### Participants

The review considered literature including information on current physical assessment tools used in clinical settings, targeting human adult population above 50 years of age, presenting with thumb base OA. We selected the term thumb base OA as it refers to a defined hand OA phenotype. The assessment selection included however the entire thumb chain components when presented in connection to thumb base OA. Documents referring to pre-existing conditions that were known to influence joint morphology or kinematics, such as traumatic injury, thumb or hand surgery, inflammatory arthritis, metabolic bone disease were excluded.

#### Concept

Extracted clinical tests of the thumb and its kinematic chain were mapped to underline their respective affinities to the thumb-specific dimensions namely architectural, ligamentous, biomechanical, neuromuscular, and proprioceptive.

#### Settings

Studies conducted in orthopedic out-patient clinics, hand therapy clinics, occupational and physical therapy outpatient clinics, rehabilitation clinics, universities, and hospitals.

#### Types of studies

Experimental and quasi-experimental study designs including randomized controlled trials, non-randomized controlled trials, before and after studies and interrupted time-series studies, analytical observational studies including prospective and retrospective cohort studies, case-control studies and analytical cross-sectional studies, descriptive observational study designs including case series, individual case reports and descriptive cross-sectional studies.

### Search strategy

The search strategy aimed to locate both published and non-published studies. An initial limited search of MEDLINE (Pubmed) and CINAHL was undertaken to identify articles on the topic. The text words contained in the titles and abstracts of relevant articles, and the index terms used to describe the articles were used to develop a full search strategy. The search strategy, including all identified keywords and index terms, was adapted for each included information source, and restricted to articles published in English and French. The reference lists of all studies selected for critical appraisal were screened for additional studies. For each database, we used words and expressions from controlled vocabulary (MeSH, EMTREE and others) and free-text searching. The search combined words and expressions of four conceptual groups: Trapeziometacarpal (TMC) joint, OA, physical examination, and different aspects of thumb stability and mobility including kinematics, kinematic chain, biomechanics, ligament, strength, neuromuscular control, and proprioception.

### Information sources

Six databases were searched from inception until May 2020 (Appendix 1). They included MEDLINE (Pubmed), CINAHL, EMBASE, and Scopus. Sources of unpublished studies and gray literature included Google Scholar, and Clinical Trials.gov.

### Study selection

Following the search, all identified citations were collated and uploaded into Endnote X9 version 3.1 and duplicates removed. Titles and abstracts were then screened by two independent reviewers for assessment against the inclusion criteria for the review. Potentially relevant studies were retrieved in full and their citation details imported into the Joanna Briggs Institute System for the Unified Management, Assessment and Review of Information (JBI SUMARI) (Joanna Briggs Institute, Adelaide, Australia).^22^ The full text of selected citations was assessed in detail against the inclusion criteria by two independent reviewers. Any disagreements that arose between the reviewers at each stage of the study selection process was resolved through discussion, or with a third reviewer. The results of the search were presented in a Preferred Reporting Items for Systematic Reviews and Meta-analyses (PRISMA) flow diagram.^24^

### Data extraction

Data were extracted from papers included in the scoping review by two independent reviewers using a data extraction tool developed by the first author. Any disagreements that arose between the reviewers was resolved through discussion, or with a third reviewer. The third reviewer, who speaks French, served as one of the main two reviewers for the French documents. Authors of papers were contacted to request additional data, when required. The two reviewers further organized the extracted tests and measures based on their respective evaluative focus of this global joint disorder^25^ relative to architecture, ligaments, biomechanics, neuromuscular control, and proprioception. Although most instruments' ability to isolate single contributors to the thumb's performance is limited by the inherent simultaneous activation of many systems, certain tests and measures have greater affinity toward some of them. We classified the affinity based on the thumb dimensions definition/affinity criteria presented in Table 1.

**Table 1.** Thumb dimensions definition/affinity criteria.

Thumb dimensions	Definition/Affinity criteria
Architectural	Relating to the basic structural form especially of bodily part.^37^
Ligamentous	Relating to tough fibrous band of tissue connecting the articular extremities of bones in place.^38^
Biomechanical	Relating to the application of the principles of mechanics to living systems particularly those living systems that have coordinated movements.^39^
Neuromuscular	Jointly involving nervous and muscular elements.^40^
Proprioceptive	Relating to subsystem of the somatosensory system that also includes pain, touch, and temperature sensation from the skin and musculoskeletal structures. It is the body’s own sense of position and motion, which includes body segment static position, displacement, velocity, acceleration, and muscular sense of force or effort.^41^

## Results

**Figure 1.**
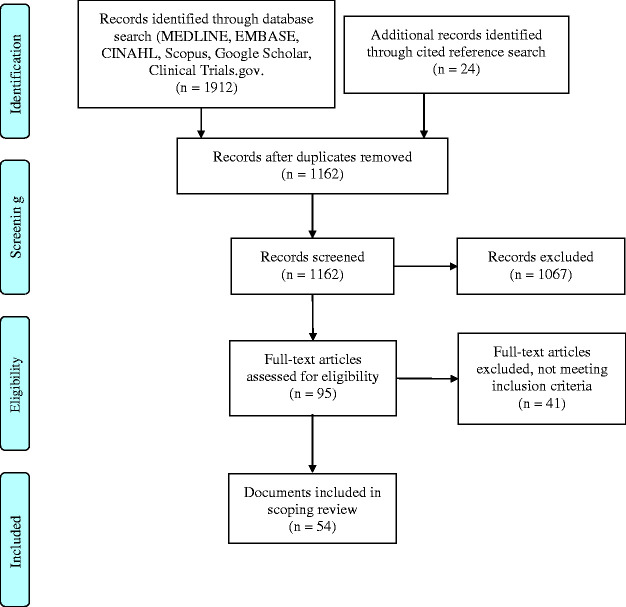
PRISMA flow diagram of search and study selection process (adapted from Moher et al.^24^).

The initial search identified 1936 documents, 1912 from different databases and 24 from extended search of cited references. Following the removal of duplicates and screening of titles and abstracts, 95 documents were selected for full text assessment. Forty-one of them did not meet the selection criteria, leaving 54 suitable for inclusion (Figure 1).

### Characteristics of included studies

Fifty-four documents containing information on clinical physical tests and measures of thumb base OA were retained for analysis. Data regarding the participants, concept, context, type of evidence source, applied tests/instruments, and psychometric validation were extracted (Characteristics of Included Studies can be found in the online Supplementary file I). The types of evidence source included systematic reviews, randomized control trials, test re-test study, cross-sectional study, prospective comparative pre-experimental study, cross-control study, prospective case control study, case-control study, prospective within patient repeated measures study, prospective cohort study, cross-sectional observational pilot study, practice guidelines, clinical trial recommendations, textbook chapter, expert opinion, and poster presentation. Some applied tests and measures were not considered in the final compilation in accordance with our selection criteria. The rejected tests and rationales are listed in Table 2.

**Table 2.** Rejected tests.

Rejected measure	Rationale
1. Observation/Inspection/Examination	Qualitative information
2. Palpation of first web space contracture	Qualitative information
3. Assessment of STT joint	No details provided
4. FIHOA	Questionnaire
5. Tenderness of hand joints	Not basal thumb OA specific
6. Ritchie articular index	Not basal thumb OA specific
7. Hand stiffness (NRS 0–10)	Subjective measure
8. Global assessment of disease activity affecting ADLs (NRS 0–10)	Subjective measure
9. Strength Dexterity test	Not clinically available
10. Pressure pain threshold (algometry)	Separate domain of assessment
11. Grip Force Control	Not clinically available
12. Rapid alternate movements	Not involving the thumb
13. Number of hand joints with limited mobility while making a fist	Not basal thumb OA specific
14. Flexion deficit of the 2nd to 5th fingers	Not basal thumb OA specific
15. Number of hand joints with bony swelling and deformities	Not basal thumb OA specific
16. Global assessment of improvement	Subjective measure
17. Functional ROM of shoulder and elbow	Not basal thumb OA specific
18. Differential Dx tests (Finkelstein’s test, Eichhoff test, peripheral nerve entrapment tests)	Not basal thumb OA specific

STT: scaphotrapeziotrapezoid; FIHOA: Functional Index for Hand Osteoarthritis; OA: osteoarthritis; NRS: numeric rating scale; ADLs: activities of daily living; ROM: range of motion; Dx: diagnosis.

### Review findings

#### Current clinical physical tests of thumb base OA

From the 54 analysed documents, 52 clinical physical tests and measures for the evaluation of thumb base OA were extracted (Table 3). The most reported measures were pinch strength (n = 27), grip strength (n = 24), and Grind test (n = 18), followed by tenderness on palpation (n = 16), range of motion (n = 13), the Modified Kapandji Index (n = 4), and the Hand Function Index (HFI) of the Keitel Function Test (n = 4). The Crank test, Distraction test, NK dexterity board, Grip Ability Test (GAT), and Button test followed (n = 3). The Thumb adduction test, Thumb extension test, Traction-shift test, Instability test, Lever test, Hand Mobility in Scleroderma (HAMIS), 2-Point discrimination test, Green test, Upper Extremity Performance Test for Elderly (TEMPA), and Sollerman test, were each reported twice (n = 2). The most recorded functional tests were the Arthritis Hand Function Test (AHFT) (n = 7), the Moberg Pick-Up Test (MPUT) (n = 7), the Jebsen-Taylor Hand Function Test (JTHFT) (n = 6), and the Purdue Pegboard (n = 5). The remaining 26 tests and measures were each mentioned in a single paper.

**Table 3.** Clinical tests and measures for thumb base OA grouped by affinity to thumb dimensions.

Thumb dimensions	Applied tests/ instruments	Specifications	Validation metrics	No. of sources applied
Architectural	Pain/tenderness on palpation	Palpation of trapeziometacarpal joint, scaphotrapeziotrapezoid joint, metacarpophalangeal joint, A1 pulley, radioscaphoid joint, first dorsal compartment.^2,7,42–55^	Yes	16
Architectural	Grind test	Axial compression and rotation of the thumb metacarpal. Positive if there is pain with or without crepitus. Axial compression applied to the thumb, whilst simultaneously moving the thumb into flexion, extension, and circumduction (Positive if elicits pain at the base of the thumb).^42–54,56–60^	Yes	18
Architectural	Metacarpal Base Compression Test	Metacarpal head is elevated with one hand and the base is depressed volarly with the other, shearing the base against the trapezium, painful in more advanced stages of disease.^47^	Not provided	1
Architectural	Crank Test	Axial loading during flexion and extension at the trapeziometacarpal joint. Maneuver reproduces pain and crepitus in the diseased joint.^48,51,52^	Not provided	3
Architectural, Ligamentous	Thumb adduction test	Firm adduction force downward onto the patient’s metacarpal head. Positive test recreates pain at the trapeziometacarpal joint.^46,60^	Yes	2
Architectural, Ligamentous	Thumb Extension Test	Firm extension force to end range. Positive test recreates pain at the trapeziometacarpal joint.^46,60^	Yes	2
Architectural, Ligamentous	Joint subluxation test	Gentle force applied to the trapeziometacarpal joint to sublux it; the examiner determines whether this elicits a pain response. Crepitus may also be apparent.^59^	Not provided	1
Architectural, Ligamentous	Pain on opposition across the palm	Not provided^45^	Not provided	1
Architectural, Ligamentous, Neuromuscular	MCP Extension Test	Patient tries to extend the thumb while the examiner provides resistance against extension by placing 1 finger on the thumb interphalangeal joint. Reproduction of pain at the trapeziometacarpal joint is a positive finding.^49^	Yes	1
Ligamentous	Lever Test	Grasping the first metacarpal just distal to the basal joint and shucking back and forth in radial and ulnar directions to their end point. Reproduction of pain at the trapeziometacarpal joint is a positive finding.^49,60^	Yes	2
Ligamentous	Traction-Shift test	Longitudinal traction to the thumb, alternate dorsal and palmar pressure over the base of the metacarpal applied to provoke subluxation and then relocation of the joint. Positive if elicits pain within the joint.^43,56^	Yes	2
Ligamentous	Laxity assessment (TMC)	Relates to how many millimeters of shift occur when the joint is passively stressed radialward. Somewhat subjective clinical assessment without radiographic confirmation. Generally, 1 mm shift = 1+ laxity, 2 mm shift= 2+ laxity, 3 mm shif t= 3+ laxity.^47^	Yes	1
Ligamentous	Painful laxity (TMC)	Sought by attempting to sublux the joint in the dorso-volar and radio-ulnar planes.^48^	Not provided	1
Ligamentous	Instability test (TMC)	Gentle pressure at the base of the metacarpal to move the metacarpal forwards and backwards on the trapezium. Detected by stabilizing the trapezium and manipulating the thumb metacarpal in a dorsal/ volar direction.^42,54^	Not provided	2
Ligamentous	Detection of CMC instability	Detected by stabilizing the trapezium and translating the metacarpal in dorso-volar and radio-ulnar way. This maneuver results in an abnormal movement of this joint.^50^	Not provided	1
Ligamentous	Degree of laxity of IP and MCP joints	Thumb IP and MCP joints laxity in both planes (hyperextension and valgus).^52^	Not provided	1
Ligamentous	MPJ instability test	Application of radially or ulnarly directed stress on the proximal phalanx while metacarpophalangeal joint in flexion.^54^	Not provided	1
Ligamentous	Screw home torque	In the final phase of opposition during either active or passive screw home torque rotation, the dorsal ligament complex tightens, the trapeziometacarpal joint is compressed, and the volar beak of the thumb metacarpal is tightly compressed into its recess area in the trapezium. This dynamic force couple changes the trapeziometacarpal joint from incongruity to congruity and from laxity to rigid stability.^26^	Not provided	1
Ligamentous	Distraction test	Rotating the thumb metacarpal base while applying axial traction, pain provocation indicates a positive test suggestive of inflamed joint capsule.^47,48,51^	Not provided	3
Biomechanical	ROM: TMC, MCP, IP, wrist joints.	Goniometric measurements of joint angles. For trapeziometacarpal joint: angle between first and second metacarpals. Compared to contralateral side.^45,48,50–52,54,61–67^	Yes	13
Biomechanical	ROM scoring system for MCP, PIP, DIP, wrist joints	Maximum active flexion and extension is recorded to the nearest 15° for each joint. Scores for each joint are assigned. The degree of abnormality is assessed on a scale of 0–3, 0 representing none or absent and 3 representing severe. A total ROM score is achieved by summing the scores of all joints of both hands.^68^	Not provided	1
Biomechanical	Thumb TMC abduction	Radial and palmar abduction measurement with the Pollexograph (large protractor measures active and passive palmar abduction as the angle between palm of the hand and tip of the thumb).^64^	Not provided	1
Biomechanical	First web space measurement	Grip size instrument including 12 transparent plexiglass cylinders, with a diameter 1–12 cm. The largest size in which the assessor could see full contact between the cylinder and the total arch of the participant's thumb and second digit.^69^	Not provided	1
Biomechanical, Neuromuscular	Modified Kanpandji Index	Thumb opposition score based on ability to touch the tip of the thumb to various positions on the fingers.^7,61,65,70^	Yes	4
Biomechanical, Neuromuscular	Hand Function Index (HFI) of the Keitel Function Test	Subset of 11 clinical tests which measure hand performance and range of motion for the right and left hands separately. Single task involving the thumb: opposition to the base of the 5th digit.^7,55,57,61^	Not with OA population	4
Biomechanical, Neuromuscular	Colditz Tear Test	Tearing of a standard piece of paper in the middle across the shortest dimension using a fingertip pinch on both hands, repeating with the stacked torn pieces of paper until unable to perform the task. Observation of the muscle recruitment and deviation from the ideal arc position.^27^	Not provided	1
Biomechanical, Neuromuscular	Hand Mobility in Scleroderma (HAMIS)	Nine tasks, assessing finger, thumb, and wrist mobility, each scored 0 (no impairment) to 3 (cannot perform), maximum score 27 per hand. Tasks involving the thumb include thumb abduction and pincer grip.^55,70^	Yes	2
Neuromuscular	Fine finger movements (FFM)	Complete thumb opposition to all fingertips in rapid succession, 3 times.^68^	Not provided	1
Neuromuscular	Grip Strength or Maximum Isometric Grip Force (MIGF)	Recorded with a hydraulic, strain gauge, or electronic dynamometer (Jamar vigorometer or Grippit, respectively).^2,7,45,51,55,60–64,66,68–80^	Yes	24
Neuromuscular	Pinch strength	Key pinch, lateral pinch and tripod pinch recorded with a Jamar dynamometer or MIE myometer. Compared to contralateral side.^2,7,45,50,51,54,55,59–64,66,68,71–74,76,77,80–85^	Yes	27
Neuromuscular	NK dexterity board/NK Hand Assessment System	Groups of manipulation tasks involving small, medium, and large objects. Each test is performed three times and the score is the average time taken to remove and replace all the objects.^61,63,74^	Not with OA population	3
Neuromuscular	Box and Blocks Test (BBT)	Consists of moving blocks with one hand, one at a time, from one compartment of a box to another that is separated by a divider. The dependent variable is the number of blocks transported in 1 min.^73^	Yes	1
Neuromuscular	Nine-Hole Peg Test (NHPT)	Consists of timed manipulation of narrow pegs from a shallow trough to holes on a board and back.^73^	Yes	1
Neuromuscular	Combined thumb abduction and index finger extension strength	Strength test performed with a Psytech Finger Flexion/Extension gauge.^67^	Yes	1
Neuromuscular	Abductor pollicis brevis muscle strength test	Not provided^45^	Not with OA population	1
Neuromuscular, Proprioceptive	Moberg Pick Up Test (MPUT)	Timed pick up of 12 small objects (randomly arranged on a wooden surface) and placement into a container positioned on the opposite side of the tested hand (dominant). Two conditions: with eyes open (MPUT-EO), to evaluate precision grip; and with eyes closed (MPUT-EC), to assess functional performance of the hand based on the proprioception of the upper limb and the tactile inputs of the fingertips.^7,55,66,69,75,77,78^	Yes	7
Proprioceptive	Light touch sensation	Assessed in the normal clinical manner for abnormalities of light touch.^68^	Not provided	1
Proprioceptive	Proprioception	Assessed in the normal clinical manner for abnormalities of proprioception.^68^	Not provided	1
Proprioceptive	Stereognosis	Assessed in the normal clinical manner for abnormalities of stereognosis.^68^	Not provided	1
Proprioceptive	Two-Point Discrimination Test	Using Weber compass or digital sliding caliper. Two points lightly touch the finger on the longitudinal axis of the radial or ulnar side of the digit. The patient indicates whether he/she feels one or two points.^68,86^	Yes	2
Proprioceptive	Semmes-Weinstein's Monofilament Test (SWMT)	Evaluates the sensory perception thresholds of the hand and fingers. Monofilaments applied to the skin, three times at each site.^75^	Yes	1
Proprioceptive	Joint Position Sense (JPS)	Joint angle was measured using a standard clear plastic goniometer. Patient is asked to reproduce a target angle of 30° TMC abduction from a maximally abducted position with eyes closed. Difference between the target angle and the reproduced angle is used as the JPS deficit criterion value.^87^	Yes	1
Inclusive (functional)	Green Test	12 tasks consisting of manipulation of small and large objects using pulp to pulp pinch, lateral pinch, spherical grip, and cylindrical grip. Each task counts the number of items moved accurately within 15 s (except for nut and bolt within 30 s).^57,81^	Not provided	2
Inclusive (functional)	Arthritis Hand Function Test (AHFT)	11 items categorized into four sections: strength, dexterity, applied strength, and applied dexterity. Tasks include grip strength, two-point pinch, three-point pinch, nine-hole peg test, lacing shoes, fastening/unfastening buttons, safety pinning, knife/fork cutting, manipulating coins, lifting a tray, and pouring water. The first three tasks are measured in pounds, the next six in seconds, and the final two in volume.^7,55,61,88–91^	Yes	7
Inclusive (functional)	Jebsen-Taylor Hand Function Test (JTHFT)	Objective measurement of standardized tasks involving seven unilateral hand skills related to activities of daily living. Scored in seconds to complete the task using the nondominant hand first and then the dominant hand. The final score is the sum of the seven tests.^7,55,57,61,78,90^	Yes	6
Inclusive (functional)	Upper Extremity Performance Test for Elderly (TEMPA)	Nine standardized tasks representing daily activities completed unilaterally or bilaterally. Each task is scored on three criteria: speed (seconds), functional rating and task analysis (quantifies performance according to five dimensions: strength, range of motion, precision of gross movements, prehension, and precision of fine movements).^61,86^	Not with OA population.	2
Inclusive (functional)	Smith Hand Function Test	Timed unilateral grasp release tasks (5), bilateral ADL's (7), tracing and writing tasks (3).^68^	Not provided	1
Inclusive (functional)	O'Connor Test	Consists of timed placement of three pins in one hole on five rows of the board with tweezers.^71^	Not provided	1
Inclusive (functional)	Grip Ability Test (GAT)	Modification of a general test of hand function based on activities of daily living. Includes 3 timed items: Putting a sock over one hand, putting a paper clip on an envelope, and pouring water from a jug.^7,55,90^	Yes	3
Inclusive (functional)	Button Test (BT)	Standard board in which the patient is asked to unbutton 5 buttons and then button them as quickly as possible, with the score recorded in seconds.^7,55,78^	Yes	3
Inclusive (functional)	Sollerman Test of Hand Function	20 standardized activities of daily living including opening jars, turning keys, handwriting, using a knife and fork, and pouring liquids from various containers. Each measured on a four-point scale, providing a maximum score of 80 for a participant who can complete each task with the correct handgrip in less than 20 s without any pain or disability.^57,85^	Not provided	2
Inclusive (functional)	Purdue Pegboard Test	Placement of metal pins in holes on a standardized board as quickly as possible, and an assembly task in which participants combined a pin, washers, and a collar in a predefined order. The dependent variable is the number of pins and assembly correctly inserted within 30 and 60 s.^7,55,62,76,86^	Yes	5

TMC: trapeziometacarpal; CMC: carpometacarpal; IP: interphalangeal; MCP: metacarpophalangeal; MPJ: metacarpophalangeal joint; ROM: range of motion; PIP: proximal interphalangeal; DIP: distal interphalangeal; ADLs: activities of daily living.

#### Thumb dimensions considered by current clinical physical tests

Architectural: Nine tests and measures presented affinity to the architectural aspect of thumb stability. Tests included in this category provided information regarding anatomical location of the disease through pain/tenderness or crepitus provocation, and deductive assessment of the degree of articular cartilage degeneration. Psychometric validation was not available for 4 of them.

Ligamentous: Fifteen tests and measures presented affinity to the ligamentous aspect of the thumb. Tests included in this category primarily informed the observer on the subjective general laxity of the trapeziometacarpal (TMC) joint and the irritability of the joint capsule. Several different testing techniques were depicted and were mainly assessing the TMC joint, except for the Metacarpophalangeal (MCP) joint Instability Test and Degree of Laxity of Interphalangeal (IP) and MCP joints as their names indicate. The Screw Home Torque proposed a method of assessment of the integrity of the dorsal ligament complex more specifically.^26^ There was an overlap with some of these tests with those of the architectural group due to their combined detection of crepitus along with laxity. Psychometric validation was not available for 9 of the 15 tests.

Biomechanical: Nine tests and measures presented affinity for the biomechanical aspect of the thumb. Tests of this category provided information on mobility of the thumb and wrist, span of the first web space, MCP joint rigidity or hyperextension, as well as arc position and muscle recruitment of the thumb under load. The Colditz Tear Test was the only one assessing mechanical behaviour under load.^27^ Six of those tests did not provide psychometric validation, including the latter.

Neuromuscular: Thirteen tests and measures presented affinity for the neuromuscular aspect of the thumb. Information gathered from those tests included thumb and finger flexor muscles strength, simultaneous thumb abduction and index extension strength, speed of grasp and release of objects, adjustability of grasp to different size objects, precision grip, and general motor control and speed of thumb and fingers. Few of those tests overlapped with the previous category due to their simultaneous consideration of joint mobility and muscular control. Five of those tests did not provide psychometric validation.

Proprioceptive: Seven tests and measures presented affinity for the proprioceptive aspect of the thumb. Information obtained from those tests included sensory discrimination, sensory perception, as well as joint position sense of the TMC joint, and functional performance of the hand based on proprioception of the upper limb and tactile input of fingertips. Psychometric validation was not available for three of them.

Inclusive: Ten of the 52 tests were categorized as inclusive due to the functional nature of their performance and did not allow for discrimination of their relative affinity. Half of them did not report psychometric validation.

#### Gaps in the current evaluative approach of thumb base OA

The gaps and their clinical and research implications are summarized in Table 4. Data obtained from the architectural test category are limited to the location of symptoms provocation and estimation of the degenerative status of the TMC joint based on crepitus.

**Table 4.** Gaps and clinical/research implications.

Thumb dimensions	Gaps	Clinical/Research implications
Architectural	No suggestion of gaps in this category.	N/A
Ligamentous	Lack of objective information regarding ligamentous laxity.Lack of validation of screw home torque mechanism as clinical test.	Clinical ability to determine status of primary stabilizer would allow clinicians to justify use of stabilization exercises in the treatment plan versus external support alone. As muscle activation could not compensate for absence of ligamentous support, consideration of long-term solutions including surgery would be justified regardless of the degeneration status of the articular surfaces.
Biomechanical	Lack of systematic assessment of wrist motion (radiocarpal and midcarpal joints) as contributor of the thumb chain. Lack of validated tools to assess the mechanical performance of the thumb chain under load.	Global assessment of the thumb chain mobility as well as its behavior under load would support rational for more specific correction allocations. As hypermobility differs from instability, this could prevent unnecessary immobilization, such as in the selection of orthosis design.
Neuromuscular	Existing instruments measuring intrinsic muscular force have not been validated with all thumb muscles or with the basal thumb OA population. Lack of assessment of specific thumb musculature and muscular imbalance.	Assessment of individual muscle strength and overall force distribution would provide:– Rationale to tailor exercise program based on individual presentation.– Ability to quantify the effect of a given exercise program on individual muscle strength.
Proprioceptive	Lack of commercial availability of validated tool such as strength-dexterity test for clinical use.	Clinical availability of the strength-dexterity test would provide: – Information on joint stability and feed forward control beyond the static force application and behavior under load. – Additional quantitative data for the evaluation of treatment efficacy.

Data from the ligamentous test category provide us with subjective appreciation of the laxity of the thumb joint capsules, which is of questionable usefulness, as debate carries on regarding the causal relationship between laxity and development of basal thumb OA.^1,28^ TMC joint stability requires integrity of its primary stabilizing ligament, which the Screw Home Torque targets, however there is no psychometric confirmation of its validity for clinical use. Considering that the posterior ligament complex also plays an important role as proprioceptive organ, imparted by the density of its mechanoreceptors,^28^ the implication of our ability to determine its status could influence the direction of treatment.

Data from the biomechanical test category inform us primarily on kinematics of the thumb joints, however, does not encompass the broader thumb chain, which includes the wrist. Although thumb base OA is considered more biomechanically driven,^29,30^ little is recorded regarding the thumb's kinetics, or behaviour under load. The Colditz Tear test was the only one to propose a way to clinically assess this feature, nevertheless it is an observational test without quantifiable results and does not have psychometric validation data. Interestingly, four distinct functional patterns of thumb key pinch were identified in healthy subjects, which were independent from the level of strength and hyperextension availability at the MCP joint.^31^ However, these patterns have not been studied with the basal thumb OA population.

Data from the neuromuscular test category do not provide information on isolated voluntary control of individual thumb muscles. The pinch and grip strength, although appearing more precise, represent a combination of muscular actions and remain devoid of information on the quality of the execution specific to the thumb. Some instruments measuring intrinsic muscular force have been identified; however, they have not been validated with all thumb muscles or with the basal thumb OA population.^32,33^ Additionally, we are unaware of a quantifiable method for assessment of muscular imbalances which has been identified as a risk factor.^29^ Although motion capture and load cell technologies can quantify motion and force with high accuracy and repeatability in research laboratory, this technology is unavailable in the clinical setting.

Regarding the proprioceptive data, as suggested in a review of current concepts regarding the proprioception of the wrist joint, when considering the proprioceptive senses under the conscious and unconscious umbrellas, data collected from our available tests is richer in the former which includes somatosensory information such as tactile, pain, joint position sense. The neuromuscular senses reflect the unconscious control in joint proprioception and include joint stability and feed-forward control that has not yet been isolated for quantitative appreciation.^34^ The strength dexterity test proposes an instrument that provides some insight on the subject however, the instrument is not currently available for clinical use.^35^

## Discussion

Fifty-two clinical tests for the evaluation of thumb base OA were identified and classified into 5 thumb dimensions recognized as pillars of stability and mobility of the thumb, namely architecture, ligaments, biomechanics, neuromuscular control, and proprioception. Despite a large number of assessments, the information provided remains insufficient regarding quantification of ligamentous laxity, performance under load, individual thumb muscle strength, joint stability and feed-forward control, which are needed to answer questions related to therapeutic interventions efficacy. The OARSI recommendations for design and conduct of clinical trials for hand OA presented in 2015 supported the study of thumb base OA as a specific phenotype. They advocated the use of instruments that are valid, reliable, responsive to change, feasible and publicly available.^7^ Several clinical tools, in particular self-administered questionnaires meet those criteria and have been extensively studied. Conversely, the few performance-based instruments recommended for the assessment of our domains of interest i.e. physical function and hand strength, were described as not disease specific, less studied, with variable metric properties for the hand OA population, and overlapping investigated domains.^7^ Overall, performance-based and self-reported functional assessments were quoted as evaluating different domains of disability and unable to identify certain specific underlying impairments.^18^ Five years later, the results of our scoping review reflect ongoing shortcomings of validated assessments and measures of characteristics specific to thumb base OA phenotype.

Pragmatic trials measure a wide spectrum of outcomes, mostly patient-centered, whereas explanatory trials focus on measurable symptoms or markers (clinical or biological).^36^ Most evidence regarding the evaluation and treatment of basal thumb OA continues to trend toward the pragmatic end of the design spectrum. The questions answered by this design relate to the effectiveness of a given intervention in real-life routine, practice, or conditions. For example: Do thumb orthoses or exercises help decrease pain and improve function of people with basal thumb OA? In contrast, the explanatory trials answer questions related to the if and how an intervention works. For example: What is the best orthosis design for thumb base OA and why? Unfortunately, this type of inquiry requires different outcome measures that are not currently available. As it pertains to basal thumb OA phenotype, the need for measures of thumb specific parameters is evident and the development and validation of new tests and instruments is required to deepen our understanding of this debilitating condition and to improve the specificity and justification of our treatment recommendations.

## Limitations

Despite adherence to a systematic search process, eligible studies may have been inadvertently excluded due to the exclusion of surgical cases in our selection criteria. However, we think that the diagnostic process is uniform and independent from conservative or surgical management. Our selection criteria also excluded tests and measures that are not clinically available, which did not allow for consideration of tools currently in developmental stage. Mention of those is made in our results section, nevertheless the official tabulation is true to the current clinical situation. Finally, although important to the development of outcome measures, assessing methodological quality of included papers was outside the scope of this review.

## Conclusions

As the thumb basal OA emerges as a distinct hand OA subset supported by its distinct joint type, clinical burden, risk factor profile, clinical relevance of synovial inflammation and therapeutic interventions, it underlines the need for specific outcome measures in addition to the general hand OA assessments.^1^

The present scoping review has highlighted the scarcity of clinical tests and measures for basal thumb OA phenotype distinct features. We should continue to build on new ideas to translate our experimental knowledge into the applied clinical environment. It is imperative to consider the multifactorial aspect of thumb stability and mobility to better assess their contribution and analyse the impact of conservative management. Moving the research strategies toward the explanatory end of the spectrum, by developing new tests and measures to capture and selectively isolate the components of the thumb multifactorial presentation would improve the target of therapy and furthermore, provide evidence of its benefits or lack thereof.

## Supplemental Material

sj-pdf-1-hth-10.1177_17589983211002560 - Supplemental material for Clinical evaluation of thumb base osteoarthritis: A scoping review Click here for additional data file. Supplemental material, sj-pdf-1-hth-10.1177_17589983211002560 for Clinical evaluation of thumb base osteoarthritis: A scoping review by Mirka Normand, Tiffany S Tang, Jean-Michel Brismée Stéphane Sobczak in Hand Therapy

## Appendix 1: Search strategy

Search conducted on 17 May 2020.

**Table:**

Database	Search
MEDLINE (Ebscohost)	(AB “thumb base” OR “trapeziometacarpal joint” OR “carpometacarpal joint” OR Hand) AND (AB Osteoarth* OR Rhizarthr*) AND (AB Assessment OR evaluation OR measure* OR test* OR instrument OR “physical exam*” OR “performance test*”) AND (AB Kinematic* OR “kinematic chain” OR biomechanic* OR ligament* OR “neuromuscular control” OR “proprioception” OR mobility OR stability OR strength*) NOT (AB knee) NOT (AB hip) NOT (AB surgery)
