# Supplementary material for: Clinical evaluation of thumb base osteoarthritis: A scoping review
Source: Hand Ther. 2021 Mar 21;26(2):63–78. doi: 10.1177/17589983211002560 (PMC10634380; doi:10.1177/17589983211002560)
Supplement: sj-pdf-1-hth-10.1177_17589983211002560 - Supplemental material for Clinical evaluation of thumb base osteoarthritis: A scoping review [file sj-pdf-1-hth-10.1177_17589983211002560.pdf]

Supplementary table- Characteristics of included studies

Citation details	Participants	Context	Type of evidence source	Applied Instruments	Psychometric validation details
Anakwe RE and Middleton SD.[1]	n/a	United Kingdom	Guidelines	Observation,<br>Palpation,<br>Grind test,<br>Instability test	Skilled examination was poor at distinguishing between symptoms from trapeziometacarpal and Scaphotrapeziotrapezoid joints (case series of 37 patients).
ASHT.[2]	n/a	United States	Practice guidelines	Grip strength,<br>Pinch strength,<br>ROM (CMC, MCP, IP, wrist),<br>MKI,<br>AHFT,<br>HFI of KFT,<br>JTHFT,<br>NK dexterity board,<br>TEMPA,<br>Global assessment of improvement	Jamar dynamometer: Concurrent validity (r>0.94), test-retest reliability (r>0.9994). Pinch strength: Good reliability. Tip pinch less reliable in subjects with OA of the hand.<br>ROM: SEM thumb CMC palmar ABD 3.5-5.9.<br>Kapandji Index: Not been studied in persons with OA of the hand (modified scale validated with RA).<br>AHFT: Reliability established with hand OA. Interrater reliability for all items (ICC≥0.99), test-retest reliability (ICC≥0.83) except for 3 items (left hand peg board dexterity: ICC=0.76; fastening safety pins: ICC=0.78; cutting with a knife and fork: ICC=0.74).<br>Concurrent validity: moderate associations between relevant items on the AHFT and physical and instrumental activities of daily living (r=0.40 to 0.75).<br>KFT: Acceptable concurrent validity not reported in the OA population.<br>JTHFT: No evidence supporting it in the assessment of individuals with hand OA.<br>NK dexterity tests: Not been studied for the OA population. Compared to normative values, persons with OA of the hand take longer to manipulate small objects, and those with OA of CMC-1 take longer to manipulate large objects.<br>TEMPA: Reliability and validity not confirmed with OA population.
Backman C.[3]	26 adults, 23 women, mean age 67 (range 49-87 years), diagnosed with OA (2 or more involved joints in their hands)	Outpatient occupational therapy services, British Colombia, Canada.	Test-retest reliability and validity study	AHFT	Inter-rater reliability (r=O.99 or better for all AHFT items), test-retest reliability (range, r=0.74 to r=O.96). Moderate correlations for most AHFT items with physical ADL and instrumental ADL.
Backman C and Mackie H.[4]	30 adult, 21 women, mean age 57.5 years, with RA or OA (2 or more	Arthritis center, rehabilitation center, regional hospital,	Inter-rater reliability study	AHFT	Inter-rater reliability among three pairs of raters was acceptable. Inter-rater reliability range (r=0.45 to r=0.99).

	involved joints in their hands)	British Columbia, Canada.			
Baker RH, Al-Shukri J and Davis TR.[5]	n/a	United Kingdom	Lecture	Observation,<br>Palpation,<br>Grind test,<br>Traction-shift test	Grind test: Specificity (97%), sensitivity (30%).<br>Traction-shift test: Sensitivity (67%), specificity (100%).
Baron M, Dutil E, Berkson L, et al.[6]	32 adults, 26 females, mean age 76.8 (range 61-91 years). 18 subjects had hand OA.	Subsidized single senior citizens apartment building, Quebec, Canada.	Cross-sectional study	Observation,<br>ROM (shoulder, elbow),<br>ROM scoring system (MCP, PIP, DIP, wrist),<br>Light touch sensation,<br>Proprioception,<br>Stereognosis,<br>Two-point discrimination,<br>Rapid alternating movements,<br>Fine finger movements,<br>Smith Hand Function Test,<br>Grip strength,<br>Pinch strength	Not provided
Buurke J, Grady J, de Vries J, et al.[7]	10 women, mean age 67.2 (range 37-90 years) with pain and OA of the CMC-1 shown on x-ray, 3 had surgery.	Rehabilitation center, Enschede, The Netherlands.	Prospective comparative pre-experimental study with randomized cross-over design	Pinch strength,<br>Green test	Not provided
Ceceli E, Gül S, Borman P, et al.[8]	60 patients, 40 controls, mean (SD) ages 58.9 ± 4.8 years and 56.6 ± 5.8 years respectively, (range 50-80). All subjects were female with HOA.	Physical medicine and rehabilitation outpatient clinic, Ankara, Turkey.	Cross-control study	Observation,<br>ROM (fingers),<br>Grip strength,<br>Pinch strength,<br>Purdue pegboard test	Not provided
Choa RM, Parvizi N and Giele HP.[9]	60 patients, 30 controls, 30 patients had osteoarthritis of the first CMC joint.	Outpatient hand clinic, Oxford, United Kingdom.	Prospective case-control study	Grind test,<br>Traction-shift test	Grind test: Sensitivity (30%), specificity (96.7%), PPV (90%), NPV (58%).<br>Traction-shift test: Sensitivity (66.7%), specificity (100%), PPV (100%), NPV (75%).
Colditz JC.[10]	n/a	North Carolina, United States.	Expert opinion	Colditz Tear test	Not provided

Crop JA and Bunt CW.[11]	n/a	Family practice, United States.	Guidelines	Palpation, Grind test	Not provided
Duong V, Bennell KL, Deveza LA, et al.[12]	124 therapists, 90% female, 92 occupational therapists (74%) and 32 physiotherapists (26%). Experience 6 to 15 years (44%), more than 16 years (33%).	Australia	Cross-sectional survey	Palpation, ROM (thumb CMC, MCP), Grind test, Pain on opposition across the palm, Pinch strength, Grip strength, Finkelstein's maneuver, APB muscle strength test, Differential diagnostic tests	Not provided
Edmunds JO.[13]	n/a	Louisiana, United States.	Narrative review	Screw home torque	Not provided
Gelberman RH, Boone S, Osei DA, et al.[14]	129 subjects divided in 3 groups, 48 subjects in the TMC arthritis group, 36 females, mean age 62+/-6 years. Other 2 groups were radial-sided wrist and hand pain and other wrist pain.	Outpatient offices, Missouri, United States.	Cross-sectional study	Palpation, Thumb adduction test, Thumb extension test, Grind test, Eichhoff test	Thumb adduction test: Sensitivity 0.94 (CI, 0.82-0.98), specificity 0.93 (CI, 0.86-0.97), interrater reliability (K= 0.79). Thumb extension test: Sensitivity 0.94 (CI, 0.82-0.98), specificity 0.95 (CI, 0.87-0.98). Interrater reliability (K= 0.84). Grind test: Sensitivity 0.44 (CI, 0.30-0.59), specificity 0.92 (CI, 0.84-0.97), interrater reliability (K=0.31). Point tenderness at the TMC: Sensitivity 0.94 (CI, 0.82-0.98), specificity 0.81 (CI, 0.71-0.88), interrater reliability (K=0.63).
Gillis J, Calder K and Williams J.[15]	n/a	Nova Scotia, Canada.	Review	ROM (CMC-1), Grip strength, Pinch strength, NK Hand assessment system	Not provided
Glickel SZ.[16]	n/a	New York, United States.	Narrative review	Observation, Palpation, Grind test, Laxity test, Distraction test, Metacarpal base compression test	Not provided
Gomes Carreira AC, Jones A and Natour J.[17]	40 subjects, 20 controls, mean ages (62.8±8.5) and (65.1±10.1) respectively, diagnosed with idiopathic TMC joint OA of	São Paulo, Brazil.	Randomized controlled trial (cross over design)	Grip strength, Pinch strength, O'Connor test	Not provided

	dominant hand. 20 females in the study and 18 in the control.				
Gravas EMH, Tveter AT, Nossum R, et al.[18]	180 subjects, mean age 63 years, 79% women, 93% right hand dominant, referred for CMCJ OA surgical consultation.	Three different hospitals, Vinderen, Norway.	Cross-sectional study	Grip strength,<br>Pinch strength,<br>Flexion deficit (fingers 2-5),<br>ROM (thumb MCP, IP),<br>ROM (CMC with Pollexograph)	Not provided
Harrison JWK and Fahmy NRM.[19]	n/a	Stockport, United Kingdom.	Lecture	Observation,<br>Palpation,<br>ROM (thumb MCP),<br>Painful laxity,<br>Grind test,<br>Crank test,<br>Distraction test	Not provided
Hermann M, Nilsen T, Eriksen CS, et al.[20]	55 subjects from another HOA cohort study with CMC1-OA, mean age (70.5 ± 6.7 years). 30 received exercises and orthosis. 29 controls received exercises only.	Hospital, Oslo, Norway.	Randomized controlled trial	Grip strength,<br>Pinch strength	Not provided
Jha B, Ross M, Reeves SW, et al.[21]	33 subjects (54 thumbs) mean age 64.6 (range 43–80.6 years (SD) 8.82). 82% female.	Private hospital, Brisbane, Australia.	Inter-rater reliability study	ROM (thumb CMC, MCP, IP),<br>Kapandji Index	Goniometric measurements: Interrater reliability varied from low (ICC 0.128 for IP joint flexion) to excellent (ICC 0.860 for MCP joint extension).<br>Kapandji index: Inter-rater reliability was excellent (ICC 0.772–0.917).
Kloppenburg M, Bøyesen P, Visser AW, et al.[22]	n/a	The Netherlands	Expert consensus	Grip strength,<br>Pinch strength,<br>Tenderness/pain on palpation	Good sensitivity to change and construct validity were established for these measures.
Kloppenburg M, Maheu E, Kraus VB, et al.[23]	n/a	The Netherlands	Clinical trial recommen-dations	AFHT,<br>Button test,<br>Purdue Pegboard<br>GAT,<br>HFI,<br>JTHFT,<br>MPUT,<br>Grip strength,	Grip strength, pinch strength, and tenderness on palpation: Supportive evidence available for reliability, sensitivity to change and validity.<br>MKI: Metric properties not investigated with hand OA.

				Pinch strength,<br>Tenderness/pain on palpation,<br>MKI	
Kroon FPB, Damman W, Liu R, et al.[24]	Cohorts from existing studies: GARP study, 207 subjects, mean age 64.2 year (59.5-69.1), 86% female. HOSTAS study, 174 subjects, mean age 60.1 years (54.6-65.9), 82.8% female. Diagnosed with HOA.	Medical Center outpatient clinic, Leiden, The Netherlands.	Cross-sectional study	Finger to palm distance (FPD) of digits 2-5,<br>HAMIS,<br>MKI,<br>Number of hand joints with limited mobility while making a fist,<br>Number of hand joints with bony swelling and deformities,<br>Grip strength	HAMIS: Intraobserver reliability ICC 0.90 (95% CI: 0.76, 0.96); intraobserver reliability (ICCs 0.94-0.97). HAMIS was the most sensitive to change (smallest detectable difference 3.7% of maximum score, followed by MKI at 12.1%.
Lawrence EL, Dayanidhi S, Fassola I, et al.[25]	66 healthy subjects, 38 female, 28 male, 66.1 ± 11.6 years (range 45–88). 33 subjects diagnosed with CMC OA (65.8 ± 9.7 years (range 44–81).	Hand clinic, Paris, France.	Cohort study with control group	Grip strength,<br>Pinch strength,<br>Box and Blocks test,<br>Nine-Hole Peg test,<br>Strength-Dexterity test	All outcome measures have been previously shown to be reliable and valid. References provided.
MacDermid J, Grewal R and MacIntyre N.[26]	n/a	Ontario, Canada.	Narrative review	Grip strength,<br>Pinch strength,<br>NK Dexterity test	Grip: Reliability coefficient 0.95, SEM 1 kg, MDC 3 kg.<br>Key pinch: Reliability coefficient 0.98, SEM 0.3 kg, MDC 1kg.<br>Tripod pinch: Reliability coefficient 0.92, SEM 0.6 kg, MDC 2kg.<br>NK Dexterity test: Small objects reliability coefficient 0.69, SEM 10s, MDC 23s. Medium objects reliability coefficient 0.72, SEM 5s, MDC 11s. Large objects reliability coefficient 0.81, SEM 8s MDC 18s.
Magni N-E, McNair PJ and Rice DA.[27]	20 subjects with symptomatic hand OA, and 19 age-gender matched healthy controls, mean age 71.7 ± 6.9 and 70.5 ± 7.7 respectively, 15 females in the OA group and 14 in the control.	Hand clinics, Auckland University staff, and community volunteers, Auckland, New Zealand	Case-control study	2-Point discrimination test,<br>TEMPA,<br>Purdue Pegboard test	2-point discrimination test: No significant difference in threshold between the control group (M= 9.48, 95% CI: 8.66, 10.45) and the hand OA group (M= 10.31, 95% CI: 8.75, 12.44), (t_{37}= 0.9, P= 0.19).<br>TEMPA: Hand OA group was significantly slower at completing functional tasks<br>compared to controls (t_{37}= -3.28, P= 0.05).<br>Purdue assembly tasks: Participants with hand OA scored significantly lower<br>(t_{37}= 2.196, P< 0.05) compared to the controls.<br>Purdue unilateral test: No significant differences between groups (t_{37}= 1.57, P< 0.063).

Marks M, Schoones JW, Kolling C, et al.[28]	n/a	Switzerland	Systematic literature review	Grind test,<br>JTHFT,<br>Sollerman hand function test,<br>Green test,<br>HFI of KFT	Not provided
Martins Nunes P, Guimarães De Oliveira D, Aruin AS, et al.[29]	10 female subjects diagnosed with HOA, mean age 60.60 ± 6.78, and 10 age-matched control.	Hand specialist physician clinic, Santa Catarina, Brazil.	Case-control study	SWMT,<br>MPUT (eyes open and closed),<br>MIGF,<br>Grip Force Control	Validity and reliability of tests has been shown previously. References provided.
McQuillan T, Kenney D, Crisco J, et al.[30]	91 subjects from a larger NIH investigation, with thumb base pain/discomfort, 23 controls mean age 56.4 ± 7.6 and 56.1 ± 17.8 respectively. Male sex (SD) 0.5(0.5) and 0.4 (0.5) respectively.	Hand and Upper Limb Center, Hospital, California and Rhode Island, United States.	Case-control study	Pinch strength	Key pinch: Most robust association with OA diagnosis. 20% decrease in key pinch strength from the control subjects' baseline was associated with a 10% increase in the OA diagnosis (95% CI, 3%-16%; p= 0.004).
Merritt MM, Roddey TS, Costello C, et al.[31]	54 subjects (70 thumbs),<br>46 women, 8 men. Average<br>age 59.5 (range 23-91). 55 had CMC OA.	Private orthopedic clinics, Occupational medicine clinic, general community, Texas, United States	Inter-rater reliability and criterion validity study	Grind test	Moderate reliability. Accurately confirms the diagnosis of CMC osteoarthritis and identifies those who do not have CMC osteoarthritis. A negative grind test does not necessarily reflect negative radiographic evidence of thumb CMC osteoarthritis. Interrater reliability (k=0.48). For the two examiners, sensitivity was 42% and 53%, specificity was 80% and 93%, positive likelihood ratio 2.64 and 6.27, and negative likelihood ratio 0.59 and 0.62, respectively.
Miller L and Jerosch-Herold C.[32]	38 subjects with CMC OA, mean age (SD) 63±8.3 years, range 43–79) and 68% female.	Local NHS Hospital, Norwich, United Kingdom.	Prospective, within patient, repeated measures study	Pinch strength	No statistically significant differences in pain, preference, or test-retest reliability between Jamar dynamometer and MIE myometer. Test-retest reliability using a single trial of tripod pinch strength was excellent with both instruments (MIE, ICC= 0.914; Jamar ICC= 0.891).
Model Z, Liu AY, Kang L, et al.[33]	62 consecutive patients (21 male, 41 female) with basal thumb pain, mean age 62 ± 8 years (range 45-86). 121 hands were	Hand surgeon clinic, United States	Prospective cohort study	Palpation,<br>Grind test,<br>Lever test,<br>MCP extension test	Palpation: Sensitivity 0.91, specificity 0.76, PPV 0.95, NPV 0.64.<br>Grind test: Sensitivity 0.41, specificity 1.00, PPV 1.00, NPV 0.26.<br>Lever test: Sensitivity 0.82, specificity 0.81, PPV 0.95, NPV 0.49.<br>MCP extension test: Sensitivity 0.65, specificity 0.95, PPV 0.98, NPV 0.36.<br>Lever test and joint palpation best reproduce the pain caused by thumb basal joint OA.

	included in the analysis.				
Murray PM.[34]	n/a	United States	Textbook chapter	Observation,<br>Grind test,<br>Joint subluxation test,<br>Pinch strength	Not provided
Odella S.[35]	n/a	Milan, Italy.	Textbook chapter	Inspection,<br>Palpation,<br>Detection of CMC instability,<br>Grind test,<br>ROM,<br>Pinch strength	Not provided
Østerås N, Hagen KB, Grotle M, et al.[36]	130 randomized subjects from two OA cohorts (mean age 66 (SD) 9; female 90%). All hand/CMC OA.	Primary health care service clinic, Hospital, Oslo, Norway.	Randomized controlled trial	Hand stiffness,<br>Global assessment of disease activity affecting ADLs,<br>Maximal grip strength,<br>MPUT,<br>Thumb web space measurement.	Grip strength: MIC value for OA population has not previously been investigated. In patients undergoing hemodialysis and MS patients, SEM was 1.4-1.8 kg, SDC (90%) was 3.4 kg.
Ouegnin A and Valdes K.[37]	29 subjects, 58 thumbs, mean age of the CMC OA group 70.31 years (±6.9), control group 69.96 years (±6.49). 79% women in the CMC OA group and 66% in the control.	Hand rehabilitation outpatient clinics (3), Florida and Pennsylvania, United States.	Descriptive-comparative cross-sectional study	JPS	Statistically significant differences in JPS scores between CMC OA and healthy group ($t= 8.67$; $P < .001$). The effect size for the difference in means was D= 2.28. There was no statistically significant difference between the mean differences for the dominant and nondominant CMC OA thumbs for JPS acuity ($t= 0.07$; $P= .47$). The minimally clinically important difference of this measurement has not been established to date.
Polatsch DB and Paksima N.[38]	n/a	New York, United States.	Narrative review	Inspection,<br>Palpation,<br>ROM (TMC, MCP and IP),<br>Stability testing,<br>Grip strength,<br>Pinch strength,<br>Grind test,<br>Crank test,<br>Distraction test	Not provided
Poole JU and Pellegrini Jr VD.[39]	n/a	Pennsylvania, United States.	Narrative review	Inspection,<br>Degree of laxity in both planes (thumb MP/IP),<br>Palpation,	Not provided

				ROM (Thumb-index web angle),<br>Crank test,<br>Grind test,<br>Differential diagnostic tests	
Poole JL.[40]	n/a	New Mexico, United States.	Narrative review	AHFT,<br>GAT,<br>JHFT	AHFT: Interrater reliability: OA, ICC ranged from 0.99–1.0. Interrater reliability: RA and OA. Pearson's correlations ranged from 0.45–0.99. Test–retest reliability: OA. ICC ranged from 0.7–0.96. Correlations between the AHFT and self-reports of hand function were fair to good, with stronger correlation for the strength items.<br>GAT: Not sensitive enough to measure differences in patients with OA who had participated in an education program compared to a control group.<br>JHFT: Shown to discriminate between subjects with and without different physical disabilities. However, the mean times were not statistically significantly different between older subjects with OA compared to the norms reported by Jebsen et al. Responsiveness to clinical change at follow up for CMC OA (ES 0.67, SRM 0.66).
Rogers MW and Wilder FV.[41]	46 subjects mean age (SD) 75(6.7), 87% female, with radiographic and symptomatic hand OA.	Research laboratory, Florida United States.	Randomized controlled trial (Controlled crossover)	Grip strength (maximal and average),<br>Pinch strength,<br>Perdue Pegboard	Not provided
Roulot E.[42]	n/a	Paris, France.	Review	Observation,<br>Palpation,<br>Grind test	Not provided
Silva FC, Adolph SMM, Da Silva RVT, et al.[43]	6 subjects diagnosed with rhizarthrosis	São Paulo, Brazil	Poster presentation of randomized controlled trial	ROM,<br>Grip strength,<br>Pinch strength,<br>MPUT	Not provided
Silva PG, Jones A, Natour J, et al.[44]	100 subjects, 50 OA and 50 control, mean age (SD) 65.4 (7.9) and 64.5 (8.4) respectively. 50% female in both groups.	University outpatient clinic, São Paulo, Brazil.	Case-control study	MPUT,<br>Grip strength,<br>Pinch strength	MPUT: OA group presented a statistically significant difference from the control group. The OA group spent more time executing test and reported greater difficulty than the control group in performing the test.<br>Grip and pinch strength measurements showed higher values for the control group. Among all the individuals studied, there were strong correlations between Cochin and MPUT results with eyes open for the right hand ($P < .001$, $r = 12:42$) and left hand ($P = .005$, $r = 0:29$) and with eyes closed for the right hand only ($P = .005$, $r = 12:28$).

Stamm T, Mathis M, Aletaha D, et al.[45]	100 subjects with HOA. Mean ± SD age of the patients was 60.7 ± 8.7 years. Eighty-seven women (87%) and 13 men (13%).	Rheumatology outpatient clinic, Vienna, Austria.	Validity study	JTHFT,<br>MPUT,<br>Button Test,<br>Grip strength	MPUT: Showed the highest raw correlation coefficient to the JTHFT; if controlled for age, the BT had the highest correlation coefficient, whereas Grip strength still had the lowest correlation coefficient.<br>Vigorometer not as reliable as other more standardized instruments, such as the Grippit.
Tsai P and Beredjiklian PK.[46]	n/a	Pennsylvania, United States.	Review	Inspection,<br>Palpation,<br>CMC instability test,<br>Grind test,<br>MPJ instability test,<br>Pinch strength,<br>ROM (thumb CMC, MP, and IP),<br>Neurologic examination for Carpal tunnel syndrome,<br>Differential diagnostic tests	ROM: Typical range for normal CMCJ is 53°(±11°), of flexion and extension and 42°(±4°) of abduction and adduction. Range of motion of the MPJ and IPJ is highly variable between individuals, and comparison with the contralateral side is important.
Villafañe JH and Valdes K.[47]	27 subjects mean age (SD) was 81.3 ± 4.7 years (range 70-90 years). 3 male and 24 female, diagnosed with unilateral thumb CMC OA.	Physical therapy department, Sangano, Italy.	Test-retest reliability study	Pinch strength	Test-retest reliability for tip, tripod, and key pinch strength: excellent for the affected side (ICC= 0.93, 0.96, and 0.99) and (ICC= 0.91, 0.92, and 0.94) for the contralateral thumb.<br>Absolute reliability (SEM and LOA): good.<br>Mean absolute differences between the test and retest for tip, tripod, and key pinch strength: 0.06 kg, 0.04 kg and 0.01 kg for the affected side respectively, and 0.05 kg, 0.06 kg, and 0.03 kg for the unaffected side, respectively.<br>No significant differences in pinch strength between the CMC OA-affected hand compared with the non-affected hand.
Villafañe JH, Valdes K, Vanti C, et al.[48]	78 subjects with unilateral thumb CMC OA, 84.6% female (mean ± SD age 83 ± 5 years), 66% female.	Italy	Test-retest reliability study	Grip strength	Test–retest reliability: excellent for the CMC OA affected side (ICC=0.947; p= 0.001) and unaffected contralateral side (ICC=0.96; p= 0.001).<br>Absolute reliability (SEM and LOA) was good.<br>Mean absolute difference between test and retest: 0.61 and 0.54 kg for the affected right hand and contralateral left hand, respectively. The 95% limits of agreement ranged from -2.54 to 2.71 kg for the affected hand and from -2.44 to 2.77 kg for the contralateral hand.
Villafañe JH and Valdes K.[49]	77 subjects, 39 with CMC OA (mean ± SD age, 81 ± 7 years), 38 healthy subjects (mean ± SD age, 78 ± 6 years), 96% female, 28 in	Physical therapy department, Sangano, Italy.	Case-control study	PPT,<br>ROM (CMC radial abduction),<br>Combined thumb abduction and index finger extension strength.	Statistically significant differences in opening strength, and ROM measurements within the CMC OA group versus healthy group (all, P< 0.01) in the dominant right hand.<br>Minimal detectable change for CMC OA: 0.12 to 0.13 lb. for opening force, and 1.24° to 1.46° for abduction ROM measurement.

	the CMC OA group and 26 in the control.				
Visser AW, Bøyesen P, Haugen IK, et al.[50]	n/a	The Netherlands	Systematic literature search	AHFT,<br>Button test,<br>Perdue Pegboard,<br>GAT,<br>Grip strength,<br>HAMIS,<br>HFI,<br>JTHFT,<br>MPUT,<br>Pinch strength,<br>Tenderness/pain on palpation,<br>Ritchie articular index	Grip strength: Inter-/intraobserver ICC: range per hand 0.91–0.94/0.90–0.92, SDD (right, left): 2.48, 1.94.<br>Pinch strength: Inter-/intraobserver ICC: range per test/hand 0.87–0.94/ 0.89–0.96, SDD (right, left): range per test 0.40–0.54, 0.42–0.63.<br>Tenderness/pain on palpation: Inter-/intra-rater ICC of Doyle index: 0.88/range per<br>rater 0.94–0.97, Inter-/intraobserver ϰ (% agreement): 0.64/0.69 (95/96)
Wajon A and Ada L.[51]	40 subjects with TMC OA, 20 in experimental group mean ± SD age, 59.7 ± 9.0 years, 61.2 ± 12.5 years for the control group. Gender M:F ratio, 5:14 and 4:17.	Hand physiotherapy practice, Sydney, Australia.	Randomized controlled trial	Pinch strength,<br>Sollerman Test of Hand Function	Not provided
Weinstock-Zlotnick G, Lin B and Nwawka O.[52]	5 subjects (9 thumbs) in CMC1 OA group, 9 healthy subjects (9 thumbs) in the control group. Female/male ratio were 4/1 and 7/2 respectively. Age range, CMC OA (51-71 years), control (26-57 years).	Hand and Upper Extremity Center in hospital, New York, United States.	Cross-sectional, observationa l pilot study	AHFT	Differences in AHFT performances apparent in tasks demonstrating grip strength, 3PP, 2PP:<br>Grip: Left (-12.5 lbs., 95% CI -57.5, 33.3), Right (-25.8 lbs., 95% CI -55, 45).<br>3PP: Left (-7 lbs., 95% CI -15.3, 1), Right (-6.3 lbs., 95% CI -14.3, -1.2).<br>2PP: Left (-3.7 lbs., 95% CI -11.3, 0.7), Right (-3.7 lbs., 95% CI -9.7, -0.7).<br>Button board task: Average of 9.3 seconds (95% CI -3.1, 24.8) longer to complete. Nine-hole peg (left and right), fastening/unfastening safety pins, lacing shoes, manipulating coins, cutting putty, lifting tin cans, and pouring water, on par with control subjects.
Wilkens SC, Meghpara MM, Ring D, et al.[53]	n/a	Massachusetts, United States.	Narrative review	Inspection,<br>Grind test,<br>Thumb adduction test,<br>Extension provocation test,<br>Lever test,<br>Pinch strength,<br>Grip strength	Grind test: Sensitivity 30% - 50%

Ziv E, Patish H and Dvir Z.[54]	32 subjects with primary hand OA (age range: 48-89 years; mean ± SD: 70.4 ± 10.0) and 25 healthy subjects (age range 56-89 years; mean ± SD of 74.6 ± 8.4).	OT and PT University departments, Hand surgery and rehabilitation unit of medical center, Tel Aviv and Ashkelon, Israel.	Reproducibi-lity study	Grip strength, Pinch strength	2PP: Diagnosing strength changes using the 2PP test is invalid due to low reproducibility in patients with POAH.<br>POAH subjects:<br>Grip: SEM (kgf) R= 1.51, L= 1.98, CVp (%) 8.76, L= 13.08.<br>Key pinch: SEM (kgf) R= 0.36, L= 0.43, CVp (%) R= 8.39, L= 10.89<br>2pp: SEM (kgf) R= 0.46, L= 0.41, CVp (%) R= 18.59, L= 18.44<br>3pp: SEM (kgf) R= 0.37, L= 0.34, CVp (%) R= 11.59, L= 11.63.<br>Average strength deficit of 27% in grip, 24%, 32%, and 28% in key pinch, two-point and three-point pinch, respectively.

ASHT: American Society of Hand Therapist; ROM: Range of motion; TMC: Trapeziometacarpal; CMC: Carpometacarpal; MCP: Metacarpophalangeal; IP: Interphalangeal; DIP: Distal interphalangeal; AHFT: Arthritis Hand Function Test; MKI: Modified Kapandji Index; HFI: Hand Function Index; KFT: Keitel Function Test; JTHFT: Jebsen-Taylor Hand Function Test; TEMPA: Upper Extremity Performance Test for the Elderly; JPS: Joint position Sense; OA: Osteoarthritis; RA: Rheumatoid arthritis; HOA: Hand Osteoarthritis; SEM: Standard of error of Measurement; ABD: Abduction; ICC: Intraclass Correlation; ADL: Activity of daily living; PPV: Positive Predictive Value; NPV: Negative Predictive Value; CI: Confidence Interval; SD: Standard Deviation; CVp: Coefficient of variation; GAT: Grip Ability Test; SWMT: Semmes-Weinstein's Monofilament Test; MPUT: Moberg Pick-up Test; MIGF: Maximal Isometric Grip Force; NIH: National Institute of Health; SDC: Smallest Detectable Change; LOA: Limits of Agreement; PPT: Pressure Point Threshold; HAMIS: Hand Mobility in Scleroderma Test; SDD: Smallest Detectable Difference; CD: Critical Difference; POAH: Primary Osteoarthritis of the Hand; 2PP: Two Point Pinch; 3PP: Three Point Pinch; OT: Occupational Therapy; PT: Physical Therapy.

1. Anakwe RE and Middleton SD. Osteoarthritis at the base of the thumb. *BMJ (Online)* 2011; 343: 1160-1166. DOI: http://dx.doi.org/10.1136/bmj.d7122.
2. ASHT. Clinical Assessment Recommendations 3 rd Edition. 2009.
3. Backman C. Reliability and validity of the arthritis hand function test in adults with osteoarthritis. *Occupational Therapy Journal of Research* 1997; 17: 55-66.
4. Backman C and Mackie H. Arthritis Hand Function Test: inter-rater reliability among self-trained raters. *Arthritis Care & Research (08937524)* 1995; 8: 10-15.
5. Baker RH, Al-Shukri J and Davis TR. Evidence-Based Medicine: Thumb Basal Joint Arthritis. *Plastic and reconstructive surgery* 2017; 139: 256e-266e. DOI: http://dx.doi.org/10.1097/PRS.0000000000002858.
6. Baron M, Dutil E, Berkson L, et al. Hand function in the elderly: relation to osteoarthritis. *The Journal of rheumatology* 1987; 14: 815-819.

7. Buurke J, Grady J, de Vries J, et al. Usability of thenar eminence orthoses: report of a comparative study. *Clinical Rehabilitation* 1999; 13: 288-294.
8. Ceceli E, Gül S, Borman P, et al. Hand function in female patients with hand osteoarthritis: relation with radiological progression. *Hand (New York, NY)* 2012; 7: 335-340. DOI: 10.1007/s11552-012-9434-0.
9. Choa RM, Parvizi N and Giele HP. A prospective case-control study to compare the sensitivity and specificity of the grind and traction-shift (subluxation-relocation) clinical tests in osteoarthritis of the thumb carpometacarpal joint. *The Journal of hand surgery, European volume* 2014; 39: 282-285. DOI: http://dx.doi.org/10.1177/1753193413508714.
10. Colditz JC. Dynamic loading posture of the thumb: The Colditz Tear Test. *Journal of Hand Therapy* 2013; 26: 360-362. Article. DOI: 10.1016/j.jht.2013.05.003.
11. Crop JA and Bunt CW. Doctor, my thumb hurts. *Journal of Family Practice* 2011; 60: 329-332.
12. Duong V, Bennell KL, Deveza LA, et al. Attitudes, beliefs and common practices of hand therapists for base of thumb osteoarthritis in Australia (The ABC Thumb Study). *Hand Therapy* 2018; 23: 19-27. DOI: http://dx.doi.org/10.1177/1758998317731437.
13. Edmunds JO. Current Concepts ofthe Anatomy ofthe Thumb Trapeziometacarpal Joint. *J Hand Surg* 2011; 36: 170-182.
14. Gelberman RH, Boone S, Osei DA, et al. Trapeziometacarpal Arthritis: A Prospective

Clinical Evaluation of the Thumb Adduction and Extension Provocative Tests. *J Hand Surg Am* 2015; 40: 1285-1291.
15. Gillis J, Calder K and Williams J. Review of thumb carpometacarpal arthritis classification, treatment and outcomes. *The Canadian journal of plastic surgery = Journal canadien de chirurgie plastique* 2011; 19: 134-138.
16. Glickel SZ. Clinical assessment of the thumb trapeziometacarpal joint. *Hand Clinics* 2001; 17: 185-195.
17. Gomes Carreira AC, Jones A and Natour J. Assessment of the effectiveness of a functional splint for osteoarthritis of the trapeziometacarpal joint of the dominant hand: A randomised controlled study. *J Rehabil Med* 2010; 42: 469–474.
18. Gravas EMH, Tveter AT, Nossum R, et al. Non-pharmacological treatment gap preceding surgical consultation in thumb carpometacarpal osteoarthritis- A cross-sectional study. *BMC Musculoskeletal Disorders* 2019; 20: 180. DOI: http://dx.doi.org/10.1186/s12891-019-2567-3.
19. Harrison JWK and Fahmy NRM. (ii) Management of peri-trapezial osteoarthritis. *Current Orthopaedics* 2005; 19: 190-195. DOI: http://dx.doi.org/10.1016/j.cuor.2005.02.015.
20. Hermann M, Nilsen T, Eriksen CS, et al. Effects of a soft prefabricated thumb orthosis in carpometacarpal osteoarthritis. *Scandinavian Journal of Occupational Therapy* 2014; 21: 31-39. DOI: https://doi.org/10.3109/11038128.2013.851735.
21. Jha B, Ross M, Reeves SW, et al. Measuring thumb range of motion in first carpometacarpal joint arthritis: The inter-rater reliability of the Kapandji Index versus goniometry. *Hand Therapy* 2016; 21: 45-53.
22. Kloppenburg M, Bøyesen P, Visser AW, et al. Report from the OMERACT Hand Osteoarthritis Working Group: Set of Core Domains and Preliminary Set of Instruments for Use in Clinical Trials and Observational Studies. *Journal of Rheumatology* 2015; 42: 2190-2197. DOI: 10.3899/jrheum.141017.
23. Kloppenburg M, Maheu E, Kraus VB, et al. OARSI Clinical Trials Recommendations: Design and conduct of clinical trials for hand osteoarthritis. *Osteoarthritis and cartilage* 2015; 23: 772-786. DOI: 10.1016/j.joca.2015.03.007.

24. Kroon FPB, Damman W, Liu R, et al. Validity, reliability, responsiveness and feasibility of four hand mobility measures in hand osteoarthritis. *Rheumatology* 2018; 57: 525-532. DOI: 10.1093/rheumatology/kex438.
25. Lawrence EL, Dayanidhi S, Fassola I, et al. Outcome measures for hand function naturally reveal three latent domains in older adults: strength, coordinated upper extremity function, and sensorimotor processing. *Frontiers in aging neuroscience* 2015; 7: 108. DOI: 10.3389/fnagi.2015.00108.
26. MacDermid J, Grewal R and MacIntyre N. Using an evidencebased approach to measure outcomes in clinical practice. *Hand Clinics* 2009; 25: 97-111.
27. Magni N-E, McNair PJ and Rice DA. Sensorimotor performance and function in people with osteoarthritis of the hand: A case-control comparison. *Seminar in Arthritis and Rheumatism* 2018; 47: 676-682.
28. Marks M, Schoones JW, Kolling C, et al. Outcome measures and their measurement properties for trapeziometacarpal osteoarthritis: a systematic literature review. *The Journal of hand surgery, European volume* 2013; 38: 822-838.
29. Martins Nunes P, Guimarães De Oliveira D, Aruin AS, et al. Relationship between hand function and grip force control in women with hand osteoarthritis. *Journal of Rehabilitation Research & Development* 2012; 49: 855-865. DOI: 10.1682/JRRD.2011.06.0117.
30. McQuillan T, Kenney D, Crisco J, et al. Weaker Functional Pinch Strength Is Associated With Early Thumb Carpometacarpal Osteoarthritis. *Clinical Orthopaedics & Related Research* 2016; 474: 557-561. DOI: 10.1007/s11999-015-4599-9.
31. Merritt MM, Roddey TS, Costello C, et al. Diagnostic Value of Clinical Grind Test for Carpometacarpal Osteoarthritis of the Thumb. *J HAND THER* 2010; 23: 261-268.
32. Miller L and Jerosch-Herold C. Intra-tester reliability of a single trial of pinch strength in patients with osteoarthritis of the first carpometacarpal joint. *Hand Therapy* 2013; 18: 17-23. Article. DOI: 10.1177/1758998313484672.
33. Model Z, Liu AY, Kang L, et al. Evaluation of Physical Examination Tests for Thumb Basal Joint Osteoarthritis. *HAND* 2016; 11: 108-112. DOI: 10.1177/1558944715616951.
34. Murray PM. Treatment of the Osteoarthritic Hand. *Green's Operative Hand Surgery E-Book* 2016: 345.
35. Odella S. *Trapeziometacarpal Joint Osteoarthritis: Diagnosis and Treatment*. Springer, 2018.
36. Østerås N, Hagen KB, Grotle M, et al. Limited effects of exercises in people with hand osteoarthritis: results from a randomized controlled trial. *Osteoarthritis and Cartilage* 2014; 22: 1224-1233.
37. Ouegnin A and Valdes K. Joint position sense impairments in older adults with carpometacarpal osteoarthritis: A descriptive comparative study. *Journal of hand therapy* 2019: 1-5. DOI: 10.1016/j.jht.2019.01.006.
38. Polatsch DB and Paksima N. Basal joint arthritis: Diagnosis and treatment. *Bulletin of the NYU Hospital for Joint Diseases* 2006; 64: 178-184. Review.
39. Poole JU and Pellegrini Jr VD. Arthritis of the thumb basal joint complex. *Journal of Hand Therapy* 2000; 13: 91-107. DOI: http://dx.doi.org/10.1016/S0894-1130(00)80034-4.
40. Poole JL. Measures of hand function: Arthritis Hand Function Test (AHFT), Australian Canadian Osteoarthritis Hand Index (AUSCAN), Cochin Hand Function Scale, Functional Index for Hand Osteoarthritis (FIHOA), Grip Ability Test (GAT), Jebsen Hand Function Test (JHFT), and Michigan Hand Outcomes Questionnaire (MHQ). *Arthritis Care and Research* 2011; 63: S189-S199. Article. DOI: 10.1002/acr.20631.

41. Rogers MW and Wilder FV. Exercise and hand osteoarthritis symptomatology: A controlled crossover trial. *J Hand Therapy* 2009; 22: 10-18.
42. Roulot E. Trapeziometacarpal osteoarthritis. *Revue du Praticien* 2008; 58: 707-715.
43. Silva FC, Adolph SMM, Da Silva RVT, et al. HPR comparison of the effectiveness of functional and night splint for rhizarthrosis: One-year follow-up of a controlled, randomized, blinded clinical trial. *Annals of the Rheumatic Diseases* 2017; 76: 1478-1479. DOI: http://dx.doi.org/10.1136/annrheumdis-2017-eular.5590.
44. Silva PG, Jones A, Natour J, et al. Moberg Picking-Up Test in patients with hand osteoarthritis. *Journal of Hand Therapy* 2017; 30: 522-528. DOI: 10.1016/j.jht.2016.10.005.
45. Stamm T, Mathis M, Aletaha D, et al. Mapping hand functioning in hand osteoarthritis: comparing self-report instruments with a comprehensive hand function test. *Arthritis and rheumatism* 2007; 57: 1230-1237.
46. Tsai P and Beredjiklian PK. Physical Diagnosis and Radiographic Examination of the Thumb. *Hand Clinics* 2008; 24: 231-237. Review. DOI: 10.1016/j.hcl.2008.03.004.
47. Villafañe JH and Valdes K. Reliability of pinch strength testing in elderly subjects with unilateral thumb carpometacarpal osteoarthritis. *Journal of Physical Therapy Science* 2014; 26: 993-995. Article. DOI: 10.1589/jpts.26.993.
48. Villafañe JH, Valdes K, Vanti C, et al. Reliability of handgrip strength test in elderly subjects with unilateral thumb carpometacarpal osteoarthritis. *Hand (New York, NY)* 2015; 10: 205-209. DOI: 10.1007/s11552-014-9678-y.
49. Villafañe JH and Valdes K. Combined Thumb Abduction and Index Finger Extension Strength: A Comparison of Older Adults With and Without Thumb Carpometacarpal Osteoarthritis. *Journal of Manipulative & Physiological Therapeutics* 2013; 36: 238-244. DOI: 10.1016/j.jmpt.2013.05.004.
50. Visser AW, Bøyesen P, Haugen IK, et al. Instruments Measuring Pain, Physical Function, or Patient's Global Assessment in Hand Osteoarthritis: A Systematic Literature Search. *Journal of Rheumatology* 2015; 42: 2118-2134. DOI: 10.3899/jrheum.141228.
51. Wajon A and Ada L. No difference between two splint and exercise regimens for people with osteoarthritis of the thumb: A randomised controlled trial. *Australian Journal of Physiotherapy* 2005; 51: 245–249.
52. Weinstock-Zlotnick G, Lin B and Nwawka O. Clinical Assessments of Hand Function in First Carpometacarpal Osteoarthritis Do Not Appear to Correlate with Radiographic Findings. *Hospital for Special Surgery Journal* 2019; 15: 269–275. DOI: https://doi.org/10.1007/s11420-019-09705-y.
53. Wilkens SC, Meghpara MM, Ring D, et al. Trapeziometacarpal arthrosis. *JBJS reviews* 2019; 7: e8-e8.
54. Ziv E, Patish H and Dvir Z. Grip and pinch strength in healthy subjects and patients with primary osteoarthritis of the hand: a reproducibility study. *The open orthopaedics journal* 2008; 2: 86-90. DOI: 10.2174/1874325000802010086.
